# A rapid expansion of hospitals: the adaptation of multi-campus hospitals under the Chinese healthcare system

**DOI:** 10.3389/frhs.2023.1226355

**Published:** 2023-08-21

**Authors:** Xingchen Zhu, Xianliang Yan

**Affiliations:** ^1^ Office of Hospital Director, The Affiliated Hospital of Xuzhou Medical University, Xuzhou, China; ^2^ Emergency Medicine Department, The Affiliated Hospital of Xuzhou Medical University, Xuzhou, China; ^3^ The Laboratory of Emergency Medicine, School of the Secondary Clinical Medicine, Xuzhou Medical University, Xuzhou, China

**Keywords:** tertiary public general hospital, hospital expansion, new hospital campus, rural urbanization, measures

## Abstract

In recent years, China’s healthcare system has undergone significant changes to meet the increasing demands of its growing population. One notable development is the rapid expansion of hospitals, particularly the adaptation of multi-campus hospitals. These multi-campus hospitals have become increasingly popular due to the many advantages that single-campus hospitals lack, including the ability to; improve medical service quality, reduce operating costs, and facilitate the development of healthcare services in rural areas. In this study, we discuss the advantages that this type of medical facility offers and identify existing and potential problems that could hinder the development of multi-campus hospitals. Additionally, we propose appropriate solutions to mitigate these problems. Overall, we propose that there should be more communication between multi-campus hospitals and other healthcare providers.

## Background

According to 2022 statistics, there were 12,000 public hospitals in China, among which more than half were county hospitals (1). As the backbone of the country’s health care system, county hospitals provide health care services to more than 900 million people in China, accounting for over 70% of the total population (2). With the continuous development of the economy, urbanization has become more and more prevalent. However, the medical technology of county hospitals has been unable to meet the needs of the public. Tertiary public general hospitals are mainly located in old urban areas, far away from new urban areas. In addition, due to the public interest nature of China’s public hospitals, the government and researchers usually plan the size of hospitals from the perspective of patient demand or top-level design by government authorities based on the overall development needs of the city. Therefore, the Chinese government launched a construction program of public hospital branches in 2023 (3). Multi-campus hospitals have become increasingly prevalent in China, primarily because they offer several advantages to healthcare providers. Multi-campus hospitals have adapted well to the Chinese healthcare system by embracing the changes that have come about (4). One of the ways that multi-campus hospitals have adapted is through the use of advanced medical technology. Healthcare providers have embraced medical technologies such as telemedicine, which has helped to improve the quality of care provided to patients. Telemedicine involves the use of telecommunication and information technologies to provide medical advice, diagnosis, and treatment to patients.

Therefore, the Chinese government launched a construction program of public hospitals branches in 2023 (3). Multi-campus hospitals have become increasingly prevalent in China, primarily because they offer several advantages to healthcare providers.

## Advantages of multi-campus hospitals

### Adaptation of multi-campus hospitals to the Chinese healthcare system

Multi-campus hospitals have adapted well to the Chinese healthcare system by embracing the changes that have come about (4). One of the ways that multi-campus hospitals have adapted is through the use of advanced medical technology. Healthcare providers have embraced medical technologies such as telemedicine, which has helped to improve the quality of care provided to patients. Telemedicine involves the use of telecommunication and information technologies to provide medical advice, diagnosis, and treatment to patients.

### Improved medical service quality

The primary advantage of multi-campus hospitals is that they offer improved medical service quality to patients (5). This is achieved through the sharing of resources, medical skills, and expertise, which allows for the provision of better healthcare services. Multi-campus hospitals have a broader range of medical specialties, which enable patients to receive more comprehensive and better care.

### Reduction of operating costs

Multi-campus hospitals help to reduce the operating costs of healthcare providers (6). This is because they enable healthcare providers to share resources, including staff, equipment, and supplies. This reduces the need for hospitals to construct new facilities, purchase costly equipment or hire additional staff members. This, in turn, helps to reduce healthcare costs, making healthcare services more affordable to the population.

## Existing and potential problems

While they offer many advantages in terms of efficiency and cost-effectiveness, there are also potential problems associated with multi-campus hospitals in China.

### Increased risk of implementing the new hospital campus management system

Evidence has shown that the improvement of the hospital management system can effectively strengthen hospital competitiveness, and improve medical care quality and patient satisfaction (7). Tsai et al. (8) found that superior management performance and effective board monitoring were related to better healthcare quality. As of the end of September 2017, all public hospitals had carried out comprehensive reform and established a complete set of management information systems (8). In China, tertiary public general hospitals have set up a modern hospital management system to establish a reasonable performance evaluation system and the mechanism of staff motivation and salary system. Establishing a new campus requires a large number of healthcare providers, and ensuring such a large number of new medical staff adapt well to the hospital management system is a great burden. Considering the adaptability of the management system and the location of the new campus, the management mode of the new campus still needs to be examined. In order to achieve higher efficiency and effectiveness the original management team must work long hours, resulting in excessive workload and fatigue, which in turn leads to lower motivation and dissatisfaction with work. As a result, it is necessary to establish a new management team.

### Increased risk of performance management system in tertiary public general hospitals

In recent decades, performance management has been used as evidence of how well organizational objectives or goals are achieved in tertiary public general hospitals (9). Regardless of the potential benefits of performance indicators, several difficulties and disadvantages have been identified for their applications to hospital management. For medical staff in a new hospital campus, performance management differs from the healthcare providers in traditional hospital campuses, considering the smaller number of patients in a traditional hospital campus. This creates a disparity for the medical staff in the new hospital campuses. Moreover, two different performance management indicators require great efforts for hospital managers and decision makers. Moreover, two different performance management indicators required great efforts for hospital managers and decision makers.

### Increased workload and psychological stress of the medical staff

In the early stage of the establishment of a new hospital campus, patients have low trust in the medical staff in the new hospital campus, despite the support of the medical staff of the traditional hospital campus (10). The medical staff, especially those working in a new hospital campus, have enormous workload and psychological pressure. Moreover, two different performance management systems make the medical staff in the new hospital campus lack a sense of belonging. These factors cause mental health problems, such as tension and anxiety. The new hospital campus attracts more and more patients to seek medical treatment over time. This would require that medical staff to invest more effort in their daily work. However, the imperfect management system of the new hospital campus leads to relying on the traditional hospital campus, which in turn leads to an extension of decision-making time and further exacerbates the physical and mental burden on medical personnel.

### Increased burden due to unclear distribution of departments and the distance

A new hospital campus may not provide all departments, due to various reasons such as insufficient equipment. As a result, patients have no choice but to transfer to the traditional hospital campus for medical treatment. However, the distance between the traditional and new hospital campuses is too far, which greatly increases the medical treatment time for patients.

Considering the particularity of the new hospital campus: the new hospital campus is not a new hospital; it is unable to use the hospital management system for new hospitals and it is different from the traditional hospital campus. How to better develop the new hospital campus, and whether to maintain the original tertiary public general hospitals management system or to find a new management system, has continued to trouble the tertiary public general hospitals. However, there has been no study to help us understand the current status of hospital management practices for new hospital campuses.

### Measures

• The expansion of multi-campus hospitals has involved significant changes in the way hospitals are structured and operated. Typically, multi-campus hospitals consist of a central administration office that oversees a number of satellite campuses. Each campus is typically dedicated to a specific medical specialty, which enables patients to access specialized services without having to travel long distances.
• Hospitals should implement a rotation plan to facilitate the learning of clinical skills by medical staff of the new hospital campus in the traditional hospital campus. Doctors with clinical experience should be selected to serve as department directors in the new hospital campus. The adaptation of multi-campus hospitals has been challenging, requiring substantial investment in infrastructure, staff training, and technology.
• A performance management system should be used between the new hospital campus and traditional hospital campus. Medical staff in the new hospital campus need to participate in medical treatment in the traditional hospital campus.
• More attention should be paid to the physical and mental health of new medical staff. The hospital should provide access to mental health services, such as establishing an online or offline psychological counseling platform, which should be made available to medical staff in the new hospital campus.
• The new hospital campus should also provide outpatient services for all departments and inpatient departments for some departments to alleviate the medical pressure in the traditional hospital campus especially for respiratory and infectious disease departments.
• One medical treatment system should be used both in the new hospital campus and traditional hospital campus, which could ensure that the inspection results are all recognized and reduce the number and time of patient medical examinations.
• Since the distance between the new and traditional hospital campus is relatively far, commuting plans, such as scheduled bus service, should be used to facilitate the commuting of medical staff and patients between the two hospital campuses. Hospitals should create efficient and effective commuting systems between campuses, as well as ensure that patients receive consistent care across all locations.
• Additionally, hospitals should innovate to provide healthcare services remotely, using telemedicine and digital tools to provide consultations and monitor patients.

Table 1 provides annual data for patient visits, operations, discharge patients, transfer patients and incomes included in this analysis. We chose one campus of a multi-campus hospital which was established in 2016. From 2018 to 2022, annual patient visits, operations, discharge patients and incomes at the new campus continued to increase. Of note, as 2019 was the first year of the SARS-CoV-2 pandemic flu in China, health services had to adapt to the high demand for hospitalizations and reduce the number of patients visits in order to contain hospital outbreaks. We saw a decrease in income in 2019 and 2020. These data indicated that multi-campus hospitals not only reduced the pressure on tertiary public general hospitals but also are recognized by patients.

**Table 1:** Results of one campus in multi-campus hospitals.

	2018	2019	2020	2021	2022
Annual patient visits	290,300	263,300	263,000	390,900	437,400
Operations	1,807	3,893	6,879	8,520	8,442
Discharge patients	43,017	39,400	39,200	46,900	45,900
Transfer patients	1,500	1,000	1,680	1,300	1,200
Incomes (million)	682	559	658	757	726

## Conclusions

The establishment of new hospital campuses for tertiary public general hospitals is inevitable. With the passage of time, the new hospital campus will become another tertiary public general hospital in new urban areas. The development of the branch hospital did not cause a significant decline in the number of patients at the main hospital, thus showing a positive impact on overall hospital development.
